# Supplementary material for: A reduced perception of sensory information is linked with elevated boredom in people with and without attention-deficit hyperactivity disorder
Source: Commun Psychol. 2025 Mar 24;3:47. doi: 10.1038/s44271-025-00233-6 (PMC11933452; doi:10.1038/s44271-025-00233-6)
Supplement: Supplementary file 2 — Additional information [file 44271_2025_233_MOESM2_ESM.pdf]

A reduced perception of sensory information is linked with elevated boredom in people with and without attention-deficit hyperactivity disorder

Johannes P.-H. Seiler[1], Jonas Elpelt[2,3], Vsevolod Mashkov[1], Aida Ghobadi[1], Ambika Kapoor[4], Daniel Turner[4], Matthias Kaschube[2,3,*], Oliver Tüscher[4,5,6,*], Simon Rumpel[1,*]

[1] Institute of Physiology, Focus Program Translational Neurosciences, University Medical Center of the Johannes Gutenberg University Mainz, Duesbergweg 6, 55128 Mainz, Germany

[2] Frankfurt Institute for Advanced Studies, Ruth-Moufang-Straße 1, 60438 Frankfurt am Main, Germany

[3] Institute of Computer Science, Goethe University Frankfurt, Robert-Mayer-Straße 11-15, 60325 Frankfurt am Main, Germany

[4] Department of Psychiatry and Psychotherapy, University Medical Center of the Johannes Gutenberg University Mainz, Untere Zahlbacher Straße 8, 55131 Mainz, Germanymtal F

[5] Leibniz Institute for Resilience Research, Wallstraße 7, 55122 Mainz, Germany

[6] Department of Psychiatry, Psychotherapy and Psychosomatic Medicine, University Medicine Halle, Martin-Luther University Halle-Wittenberg, Halle, Germany

[*] Senior authors that contributed equally

Correspondence concerning this article should be addressed to Johannes Seiler, Institute for Physiology, Focus Program Translational Neurosciences, University Medical Center of the Johannes Gutenberg University Mainz, Duesbergweg 6, 55128 Mainz, Germany. E-mail: johseile@uni-mainz.de

- Additional information -

Supplementary Figures

Supplementary Figure 1

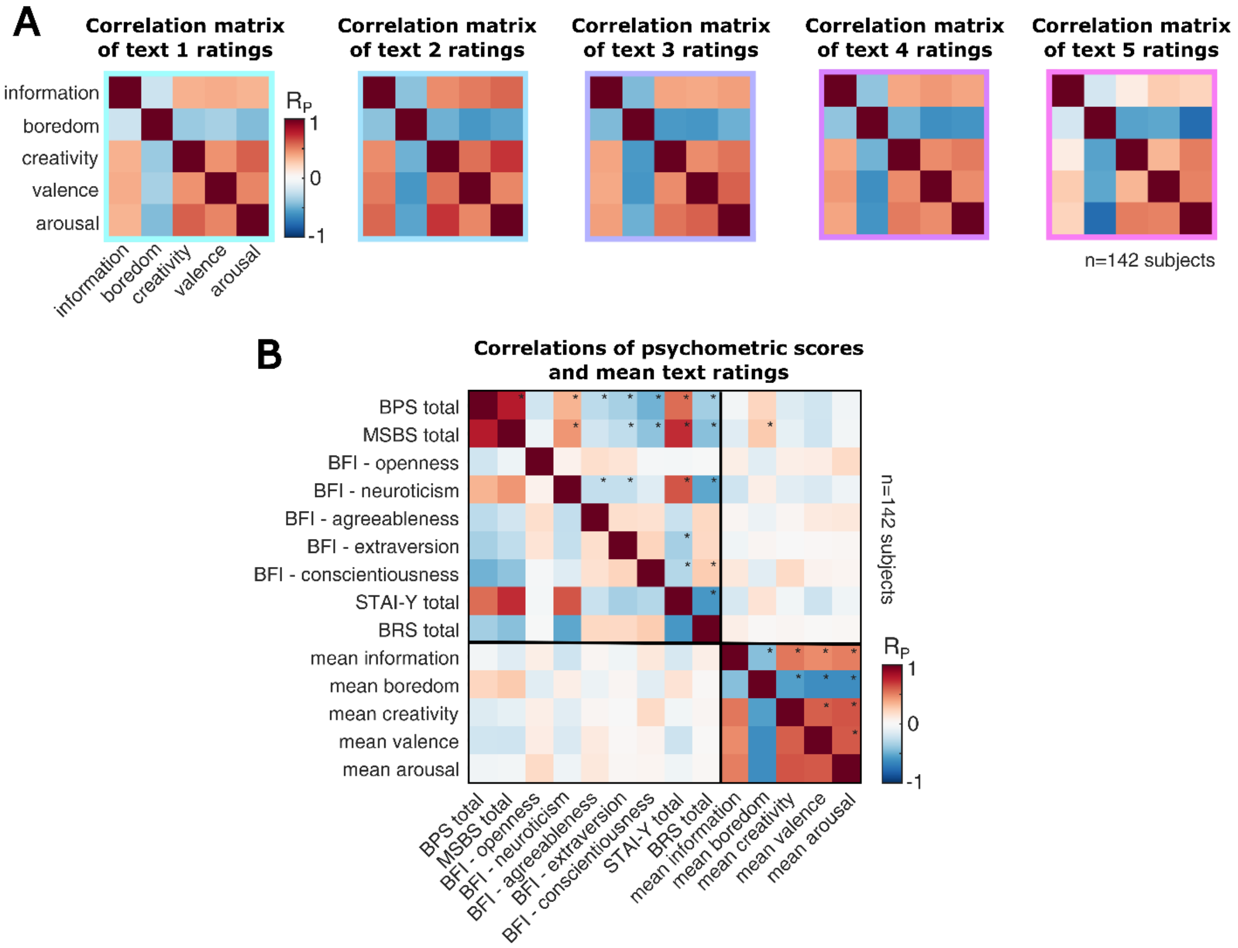

Supplementary Figure 1 – Correlations of text ratings and psychometric scores: (**A**) Correlation matrices of the rated text sentiments for the data of each text independently. The matrices show a vastly congruent pattern, only in conditions of low (text 1) and high complexity (text 5), the correlations of perceived information and the other ratings is weaker. (**B**) Correlations of the psychometric questionnaire scores and the mean text ratings (*: p<0.055, indicating statistical significance after Bonferroni correction for multiple testing). The correlations between the psychometric measures match previous studies[30,98-100,103]. State boredom during the experiment (MSBS score) is positively associated with rating the texts as more boring. The lower right part of the matrix is equivalent to Figure 3B, the correlations of mean information ratings and BFI scores are equivalent to the display in Figure 4E.

Supplementary Figure 2

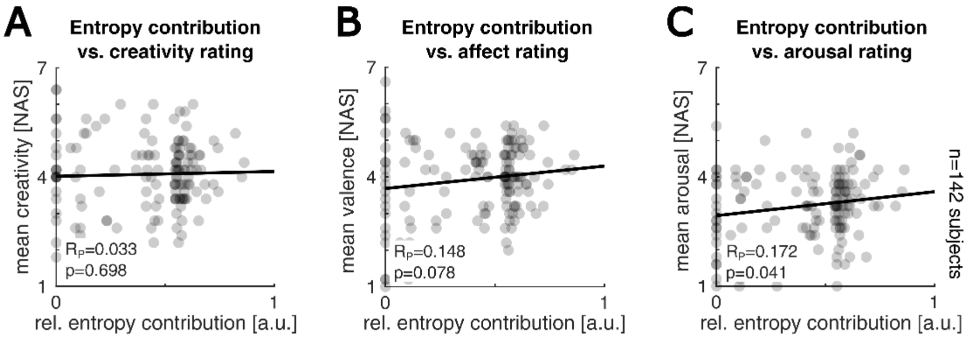

Supplementary Figure 2 – Sensitivity to external information is only weakly associated with other sentiments: (**A**) Scatter plot and Pearson correlation of each participant's mean contribution of entropy (reflecting the individual information sensitivity, see Methods) and the mean creativity rating over all texts (n=142 participants, black line indicates a linear fit). Here, no link between information sensitivity and creativity ratings is observed. (**B-C**) Equivalent plots for the mean ratings of valence (affect) and arousal, suggesting weak positive associations, in line with the negative correlation of boredom and information sensitivity (see Figure 4F).

Supplementary Figure 3

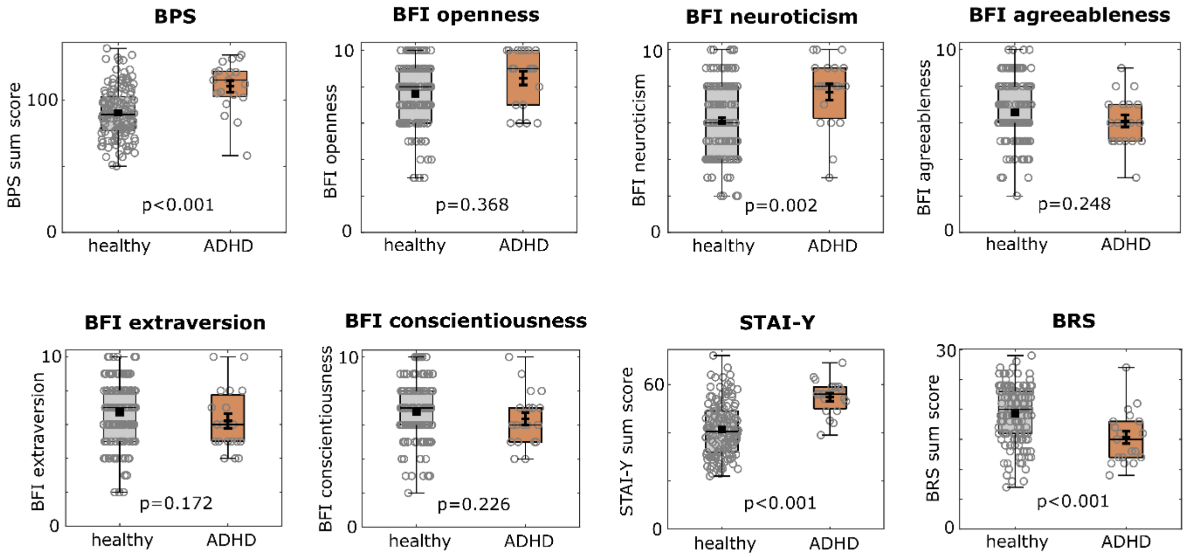

Supplementary Figure 3 – Psychometric differences between healthy control participants and ADHD patients: Psychometric self-report assessments from the healthy participants (n=142) and ADHD patients (n=19). ADHD patients show a robustly increased boredom proneness, anxiety as well as reduced mental resilience (the reported p-values refer to Wilcoxon rank sum tests; abbreviations: BPS: Boredom Proneness Scale, BFI: Big Five Inventory, STAI-Y, State Trait Anxiety Inventory, BRS: Brief Resilience Scale). Dots indicate data of single participants. Horizontal bar: median. Box: quartiles around the median. Whiskers: top and bottom quartiles.

Supplementary Figure 4

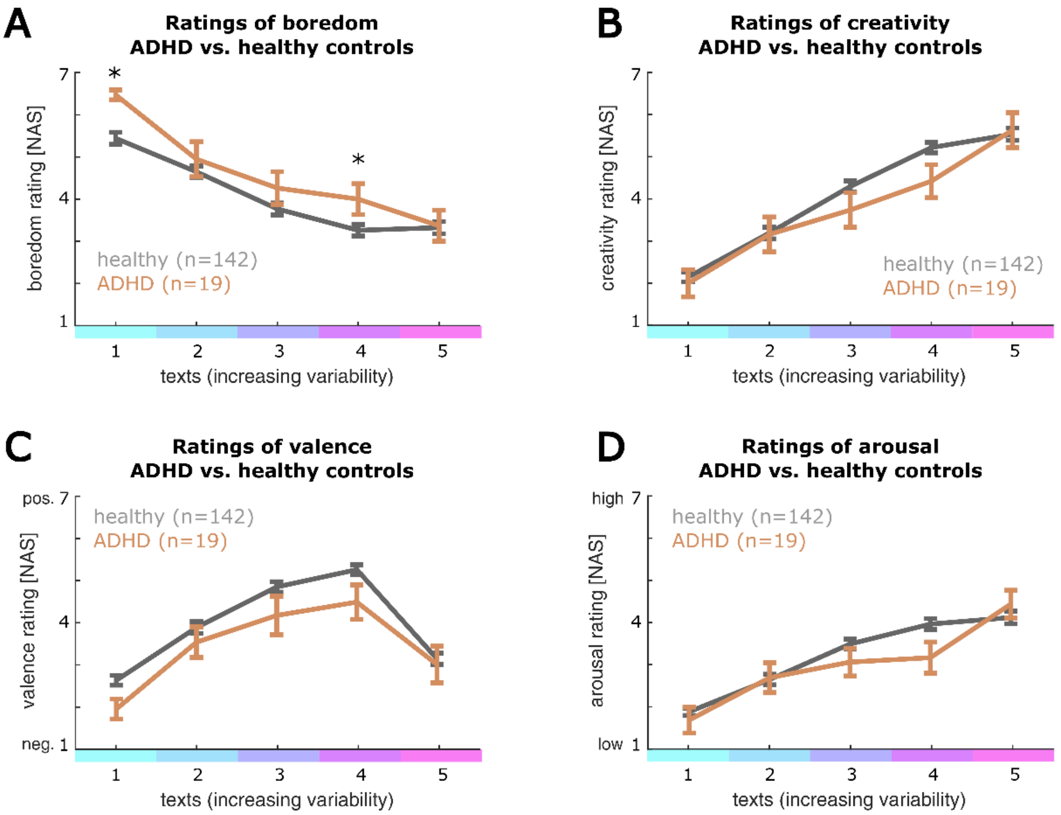

Supplementary Figure 4 – Comparison of text sentiment between healthy control participants and ADHD patients: (**A**) Ratings of boredom over texts for n=142 healthy participants versus n=19 ADHD patients (lines indicate mean, bars indicate SEM). ADHD patients tend to report higher boredom for the texts (*: $p<0.05$ in Wilcoxon rank sum test). (**B-D**) Equivalent plot for the creativity ratings, showing no significant differences for the ratings of creativity, affect and arousal (for all comparisons $p>0.05$ in a Wilcoxon rank sum test). Together, this illustrates that besides modest effects of boredom perception, text sentiment is widely comparable between healthy control participants and ADHD patients.

Supplementary Tables

German (used in experiment, each text with 100 words)	
**Text 1**	Im Winter hüllt sich die Welt in kalten Schnee. Der Winter ist kalt, der Schnee ist weiß. Die Natur im kalten Winter ist in weißen Schnee gehüllt. Ein Spaziergang durch den weißen Schnee ist kalt, der kalte Winter allgegenwärtig. Der kalte Winter hüllt die Natur in weißen Schnee. Der Winter hüllt die Welt in eine weiße Schneedecke, die kalte Schneedecke leuchtet weiß. Die Kälte des Schnees im Winter umhüllt die Natur, während man durch den Schnee spaziert. Weißer Winter, die Zeit der weißen Kälte, des weißen Schnees und der kalten Stille, die die Welt in eine weiße, kalte Schneedecke hüllt.
**Text 2**	Im Winter erstarrt die Welt unter Schnee. Die Luft ist kalt, der Schnee macht alles weiß. Die Natur ruht stumm, kahle Bäume sind in weißen Schnee gehüllt Ein Spaziergang durch die weißen Wälder ist ein kaltes Erlebnis, die kalte Stille allgegenwärtig. Der kalte Winter hüllt die verschneite Natur in weißen Glanz. Der Winter hüllt die Welt in eine weiße Schneedecke, die Augen leuchten vor Kälte und Schnee. Die weiße Kälte durchdringt jeden Atemzug, während man sich in dicke Decken hüllt. Winter, die Zeit der weißen Kälte, des weißen Schnees und der Stille, die die Welt in eine weiße Schneedecke hüllt.
**Text 3**	Im Winter erstarrt die Welt unter Eis. Die Luft ist kalt, der Schnee macht alles weiß. Die Natur ruht stumm, kahle Bäume sind von weißen Schneekristallen umgeben. Ein Spaziergang durch die weißen Wälder ist ein frostiges Erlebnis, die kalte Stille allgegenwärtig. Die klirrende Kälte hüllt die verschneite Natur in einen eisigen Glanz. Der Winter hüllt die Welt in eine weiße Schneedecke, die Augen leuchten vor Kälte und Bewunderung. Die klirrende Kälte durchdringt jeden Atemzug, während man sich in dicke Decken hüllt. Winter, die Zeit der Kälte, des Schnees und der weißen Stille, die die Welt in ein frostiges Märchenland verzaubert.
**Text 4**	Im Winter erstarrt die Welt unter Eis. Die Luft ist frisch, dicke Flocken kleiden alles weiß. Die Natur ruht stumm, kahle Bäume sind von glitzernden Schneekristallen umgeben. Ein Spaziergang inmitten der weiß gepinselten Stämme ist ein beflügelndes Erlebnis, die rauschende Stille allgegenwärtig. Der klirrende Frost hüllt Wald und Fluss in einen glänzenden Mantel. Alles ist wundersam bedeckt von einer samtigen Decke, die Augen leuchten vor Kälte und Bewunderung. Der pfeifende Frost durchdringt jeden Atemzug, während man sich in Decken träumt. Winter, die Zeit der erwärmenden Kälte, der weichen Flocken und des stillen Knisterns, die die Welt in ein Märchenland verzaubert.
**Text 5**	Im Sommer zerfällt die flauschige Galaxie in Kaugummi. Die Luft ist fett, bunte Luftballons umhüllen alles laut. Die Politik surrt wild, laute Roboter sind von glänzenden Regenbogen umgeben. Ein Tornado inmitten der neonbunten Schaltkreise ist ein schmelzendes Erlebnis, der schrille Lärm allgegenwärtig. Das dampfende Getümmel trägt Stadt und Wüste in einen schillernden Wirbel. Alles ist phantastisch bedeckt von einer knisternden Ananas, die Zehen leuchten vor Hitze und Überraschung. Der gurgelnde Vulkan durchdringt jeden Schokoriegel, während man sich in Blumen träumt. Montag, die Zeit der erfrischenden Walze, der harten Gelees und des lauten Knalls, die den Zwerg in ein Zirkuszelt verzaubert.
**English translation**	
**Text 1**	In winter, the world wraps itself in cold snow. Winter is cold, and the snow is white. Nature in the cold winter is enveloped in white snow. A walk through the white snow is cold, the cold winter omnipresent. The cold winter blankets nature in white snow. Winter wraps the world in a white blanket of snow, the cold snow blanket gleams white. The chill of the snow in winter envelops nature as you walk through the snow. White winter, the time of white cold, white snow, and cold silence that envelops the world in a white, cold blanket of snow.
**Text 2**	In winter, the world freezes under snow. The air is cold, the snow makes everything white. Nature rests silently; bare trees are enveloped in white snow. A walk through the white forests is a cold experience, the cold silence omnipresent. The cold winter envelops the snow-covered nature in white splendor. Winter blankets the world in a white snow blanket, eyes gleaming with cold and snow. The white cold permeates every breath as you wrap yourself in thick blankets. Winter, the time of white cold, white snow, and silence, enveloping the world in a white blanket of snow.
**Text 3**	In winter, the world freezes under ice. The air is cold, the snow makes everything white. Nature rests silently; bare trees are surrounded by white snow crystals. A walk through the white forests is a frosty experience, the cold silence omnipresent. The crackling cold envelops the snow-covered nature in an icy glow. Winter blankets the world in a white snow blanket, eyes gleaming with cold and admiration. The crackling cold permeates every breath as you wrap yourself in thick blankets. Winter, the time of cold, snow, and white silence, enchanting the world into a frosty fairytale land.
**Text 4**	In winter, the world freezes under ice. The air is fresh, thick flakes dress everything in white. Nature rests silently; bare trees are surrounded by glittering snow crystals. A walk amidst the white-painted trunks is an uplifting experience, the rustling silence omnipresent. The crisp frost envelops the forest and river in a gleaming coat. Everything is wonderfully covered by a velvety blanket; eyes glow with cold and admiration. The whistling frost penetrates every breath as you dream yourself into blankets. Winter, the time of warming cold, soft flakes, and quiet crackling that transforms the world into a fairyland.
**Text 5**	In summer, the fluffy galaxy disintegrates into chewing gum. The air is thick; colorful balloons envelop everything loudly. Politics hums wildly; loud robots are surrounded by shiny rainbows. A tornado amidst the neon-bright circuits is a melting experience, the shrill noise omnipresent. The steaming commotion carries city and desert into a shimmering whirl. Everything is fantastically covered by a crackling pineapple; toes glow with heat and surprise. The gurgling volcano penetrates every chocolate bar as you dream yourself into flowers. Monday, the time of refreshing rolls, hard jellies, and loud pops, enchanting the dwarf into a circus tent.

Supplementary Table 1 – Text stimuli with systematically varying complexity
